# Virtual Reality as a facilitator for social participation among persons with mental health and substance use disorders

**DOI:** 10.1192/j.eurpsy.2026.11084

**Published:** 2026-07-31

**Authors:** M. Leonhardt, J. Aasen, O. E. Flaten, A. B. Bech, L. Lien

**Affiliations:** 1 Research Centre for Substance Use Disorders and Mental Illness, Inland Hospital Trust, Brumunddal; 2 Inland University, Hamar, Norway

## Abstract

**Introduction:**

Individuals with co-occurring mental health and substance use disorders (MHD/SUD) often face exclusion, double stigma, and challenges in their daily lives. Virtual Reality (VR) has been used in the treatment of substance use and mental health issues, but little is known about the target groups’ behaviour and attitudes towards this technology and how it may be useful in addressing marginalization.

**Objectives:**

To explore, develop, and evaluate the potential of using VR in the recovery process for individuals with MHD/SUD to enhance their social participation and improve quality of life.

**Methods:**

The study is interdisciplinary and multisectoral and consists of three phases which built upon each other and follow the VR-CORE-framework: In phase 1, in-depth interviews were conducted with MHD/SUD-patients, focus groups with healthcare providers, and an interview survey with 100 MHD/SUD-patients to explore barriers and facilitators for social participation. In phase 2, we used these data to co-create with users a VR intervention, which was pre-tested among the target group. The final phase is a two-arm, statistician-blinded, pragmatic, multi-cite, randomized controlled trial (RCT), where we are testing the effect of the VR intervention versus standard treatment among MHD/SUD-patients in specialized addiction treatment. Primary outcome is general functioning, measured by the World Health Organization Disability Assessment Scale (WHODAS) 2.0 at 6 months post-intervention.

**Results:**

Based on the exploratory studies in phase 1, a VR intervention was developed in phase 2, resulting in three learning modules addressing social cognition, social communication skills, and social competence. The pre-test indicated that VR could provide an opportunity for MHD/SUD patients to practice social skills in a protected environment. The RCT is still ongoing, see CONSORT flow diagram (Figure 1).

**Conclusions:**

The expected results will have implications for the quality of life of individuals with MHD/SUD, including other marginalized groups. Offering VR may have positive effects on the delivery of healthcare services to groups that are otherwise hard to reach, and it may also have economic implications by making more resources available to expand services for those in greatest need.

**Disclosure of Interest:**

None Declared

